# A Sustainable-by-Design Process for the Selective Photooxidation of Ethylbenzene in a Scalable Agitated Baffle Reactor

**DOI:** 10.1021/acsomega.5c07359

**Published:** 2025-09-06

**Authors:** Gary Morrison, Nayan Jyoti Mazumdar, Nancy Artioli, Megan Smyth, Scott Wharry, Thomas S. Moody, Jonty Thornton, Edward Bainbridge, Nikolay Cherkasov, Haresh Manyar

**Affiliations:** † School of Chemistry and Chemical Engineering, 1596 Queen’s University Belfast, David Keir Building, Stranmillis Road, Belfast BT9 5AG, U.K.; ‡ Almac Group, Department of Technology, 20 Seagoe Industrial Estate, Craigavon, Northern Ireland BT63 5QD, U.K.; § Department of Civil, Environmental, Architectural Engineering and Mathematics, University of Brescia, Via Branze, 43, 25123 Brescia, Italy; ∥ Arran Chemical Company, Unit 1 Monksland Industrial Estate, Athlone, Co. Roscommon N37 DN24, Ireland; ⊥ Stoli Chem, Prince Philip Building, Wellesbourne Campus, Warwickshire CV35 9EF, U.K.

## Abstract

This study presents a sustainable-by-design approach for the selective photooxidation of ethylbenzene under continuous flow conditions using sodium anthraquinone-2-sulfonate (SAS) as a water-soluble photocatalyst. The reaction was conducted in a scalable agitated baffle reactor (SABRe) under ultraviolet (UV)-A irradiation (365 nm), enabling enhanced mixing, illumination, and gas–liquid contact. To systematically optimize the process, a four-factor central composite design based on response surface methodology (RSM) was employed, evaluating the influence of catalyst loading, liquid and gas flow rates, and light intensity. The study revealed that oxygen mass transfer from air is a key limiting factor, which was successfully addressed by implementing counter-current gas–liquid flow and increased agitation speeds. These modifications led to a significant improvement in ethylbenzene conversion and selectivity toward acetophenone. The SABRe reactor achieved a space–time yield (STY) of 14.8 g L^–1^ h^–1^, representing a three fold improvement over the conventional microchannel reactor configuration. Under optimized conditions, an extended 8 h continuous operation processed 1.44 L of feed solution, delivering an 87% isolated yield with ≥98% product purity. The modular and scalable nature of the SABRe platform, combined with efficient process intensification strategies, underscores its potential for sustainable chemical manufacturing and future scale-up via a numbering-up approach for photocatalytic C–H functionalization using our intensified continuous flow technology.

## Graphical abstract

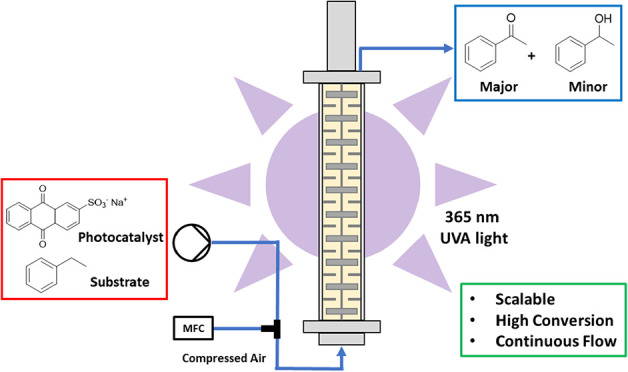

## 1 Introduction

Conventional industrial oxidation processes often rely on stoichiometric inorganic oxidants, such as dichromate and permanganate, which are toxic, hazardous, and energetically demanding. As a greener and more sustainable alternative, the direct utilization of molecular oxygen or air offers a low-cost, atom-economical route for oxidation reactions.^1^ To harness molecular oxygen from air effectively, photocatalysis has emerged as a promising tool for advanced oxidation processes, enabling the development of novel chemical transformations under mild conditions for broad reactions and substrate scope.^2–4^

Of particular interest is the application of organic photocatalysts for C–H functionalization. This strategy holds significant potential for step- and atom-economical synthesis, particularly in the late-stage modification of natural products and pharmaceutical targets.^5–8^ Compared to metal-based photocatalysts, such as those containing ruthenium or iridium, organic photocatalysts offer advantages in terms of cost, toxicity, and environmental impact.^8,9^

Building on previous studies, sodium anthraquinone-2-sulfonate (SAS) was selected as a model organic photocatalyst due to its commercial availability, low-cost, water solubility, and established photochemical activity.^10–13^ Upon photoexcitation, SAS undergoes intersystem crossing to a triplet state and facilitates hydrogen atom abstraction from substrates such as ethylbenzene, generating reactive radical intermediates that can undergo subsequent oxidation steps (Figure 1).^14^

**Figure 1:**
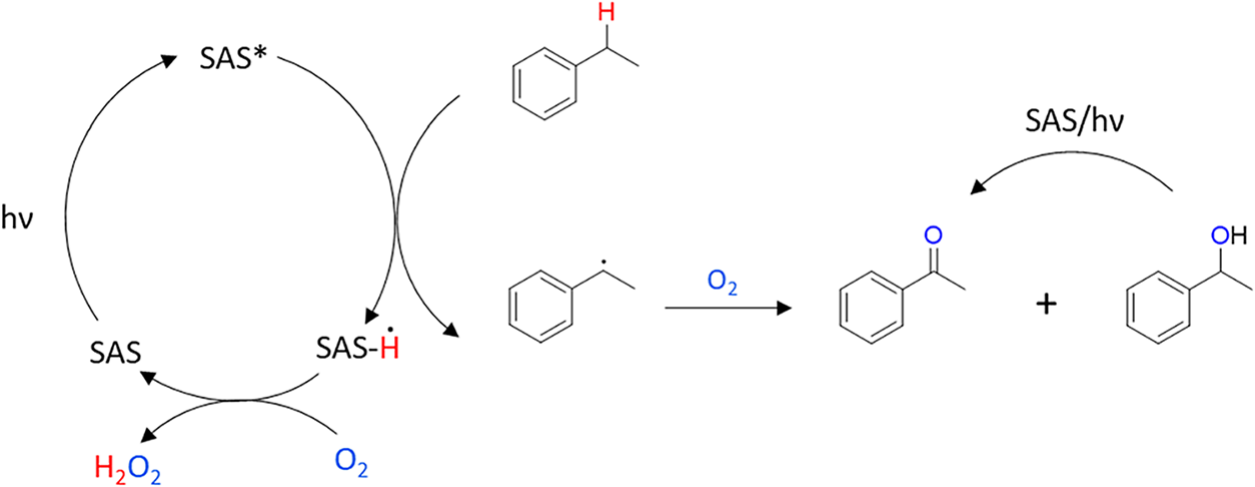
Proposed reaction mechanism of SAS oxidation of the benzylic position of ethylbenzene to acetophenone using air as a source of oxygen.

Scaling up photooxidation reactions presents several challenges. A major limitation is the reduction in interfacial area-to-volume ratio with increasing reactor size, which exacerbates the inherently low solubility of oxygen in organic solvents and results in poor gas–liquid mass transfer.^15–18^ Another limiting factor is the rapid attenuation of light through the reaction medium, governed by the Beer–Lambert–Bouguer law (eq 1, Figure 2), which further constrains the scalability. In large-volume batch reactors, limited light penetration slows reaction rates and increases the risk of overirradiation, potentially leading to the formation of undesired byproducts.

**Figure 2:**
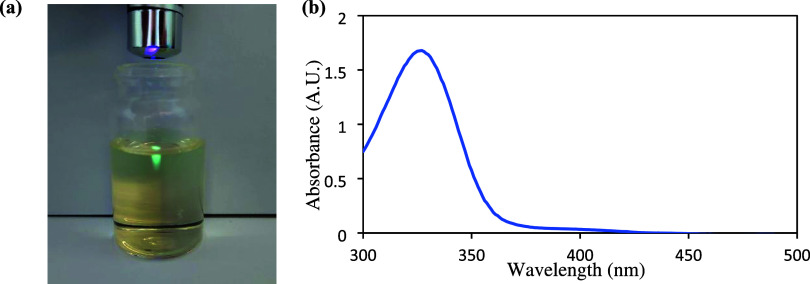
(a) Photograph demonstrating the Beer–Lambert–Bouguer law. The photograph depicts light penetration of a 405 nm laser through 10 mL of solution of 15 mol % SAS and (b) UV/vis absorbance for SAS in 75% V/V acetonitrile/water (300–500 nm).

While molecular oxygen is ideal from both cost and atom economy perspectives, its use introduces safety concerns when combined with flammable organic solvents and heat generated from light sources (e.g., lamps or light-emitting diodes (LEDs)). Moreover, the accumulation of reactive peroxide intermediates poses further risks. Addressing these challenges is essential to developing safe, efficient, and industrially viable photooxidation processes by using air instead of molecular oxygen.

Beer–Lambert–Bouguer law.

1 A=εlC

These challenges make photooxidation processes difficult to scale using conventional batch reactors, which are commonly employed in the pharmaceutical and fine chemical industries. Continuous flow technology offers a promising solution for reproducible, safe, and scalable chemical processing. The use of smaller reactor elements improves light penetration and uniformity, increases the interfacial area-to-volume ratio, and significantly enhances the gas–liquid mass transfer. Additionally, smaller reactors provide better heat dissipation and reduce the risk associated with the accumulation of reactive intermediates.^19,20^

Scalability in such systems can be achieved through a “numbering-up” approach by multiplying reactor elements or by extending manufacturing times within a single flow system.^21–24^ The scalable agitated baffle reactor (SABRe) is specifically designed with numbering-up in mind. It comprises ten continuous stirred tank reactors (CSTRs) in series, providing a total working volume of 120 mL. This modular setup offers a much higher surface-area-to-volume ratio than batch reactors, improving illumination, mixing efficiency, and temperature control.

Each reactor element also delivers high specific mixing power, ensuring rapid fluid circulation and enhancing gas–liquid mass transfer. Maintaining consistent mixing power per unit volume is critical for ensuring scale-invariant performance.^25^ The series CSTR configuration provides additional advantages, including more plug-flow-like behavior and tighter residence time control compared to a single CSTR. While the overall footprint may be larger than that of a traditional plug-flow reactor, the total volume is smaller than that of a single large CSTR. Furthermore, CSTR systems are less susceptible to fouling and blockages, making them advantageous for handling heterogeneous or solid-forming reactions that often pose challenges in tubular reactors.

For comprehensive process optimization, chemometric techniques such as response surface methodology (RSM), based on design of experiments (DoE), are widely adopted.^27^ The multivariate nature of RSM allows for the consideration of both factor interactions and nonlinear relationships between variables and responses, enabling a more holistic understanding of the experimental system. For example, RSM was applied to model the selectivity of triacetin in glycerol esterification with acetic acid, with results indicating that reaction temperature and the mole ratio of reactants had a synergistic effect on product selectivity.^28^ Further, Mazumdar et al. optimized the hydrogenation of levulinic acid to γ-valerolactone (GVL) using Cu catalysts supported on manganese oxide (OMS-2). This approach identified hydrogen pressure as the most influential variable, followed by catalyst loading, with optimal conditions (190 °C, 20 bar H_2_, 20 wt % Cu) yielding up to 98% GVL in water. The model demonstrated a combined effect of reaction temperature (°C) and H_2_ pressure (bar) on the yield of GVL.^29–31^ These examples underscore the versatility and efficiency of DoE and RSM in optimizing a wide range of chemical processes, particularly when balancing multiple variables to achieve high performance and scalability. Moreover, the RSM can elucidate underlying trends and relationships between variables, providing valuable insights that support rational process development across diverse chemical systems. In this study, the photocatalytic oxidation of ethylbenzene was investigated using RSM to evaluate the effects and interactions of four key variables: liquid flow rate, gas flow rate, catalyst loading, and number of ultraviolet (UV) lamps. Additionally, the impact of counter-current gas–liquid mixing and agitation speed on process performance was explored. These investigations enabled the development of optimized conditions for efficient and scalable C–H oxidation under continuous flow.

## 2 Experimental Section

### 2.1 Materials

All solvents were purchased from Fisher Scientific and used without further purification. Substrates and reagents were purchased from Sigma-Aldrich and used as received. Compressed air was purchased from BOC and used as received. All PTFE and PEEK parts were purchased from Cole-Parmer.

### 2.2 Experimental Setup

The reactor photographs and key dimensions are listed in Figure 3. The reactor is vertical and is surrounded by 8 lamps, Dulux L BL UVA 36W/78 2G11. Each had an electric power of 36 W and a peak wavelength of 365 nm (Figure 3a). The reactor contains 2 concentric glass tubes: (1) the inner tube is 26 mm inner diameter and 2 mm wall thickness and (2) an outer jacket tube 49 mm inner diameter and 2 mm wall thickness. The lamps were mounted in two concentric circles: 2 lamps sidewise at 220 mm diameter around the reactor center and 6 further lamps around 275 mm diameter (Figure 3b). The wall of the box was covered with reflective aluminum film and cooled with air flow to maintain the lamp temperature below 50 °C. The light went through the reactor jacket filled with circulating water to maintain the set fluid temperature. The reactor itself contains 10 equal stirred tanks (Figure 3c). Every tank was 26 mm in diameter and 25 mm in length (Figure 3d). The tank contains 4 vertical baffles 3.2 mm in diameter positioned at 20 mm circle and a Rushton impeller 15.5 mm in diameter 3 mm thick. The vertical baffles (feeding tubes) contained holes to introduce additional components with 1 mm holed positioned at the tank center. These tubes were used to introduce the liquid and fluids into the corresponding stirred tanks. The fluid was traveling exclusively at the tank center through the hole 6 mm in diameter around the 5 mm diameter shaft.

**Figure 3:**
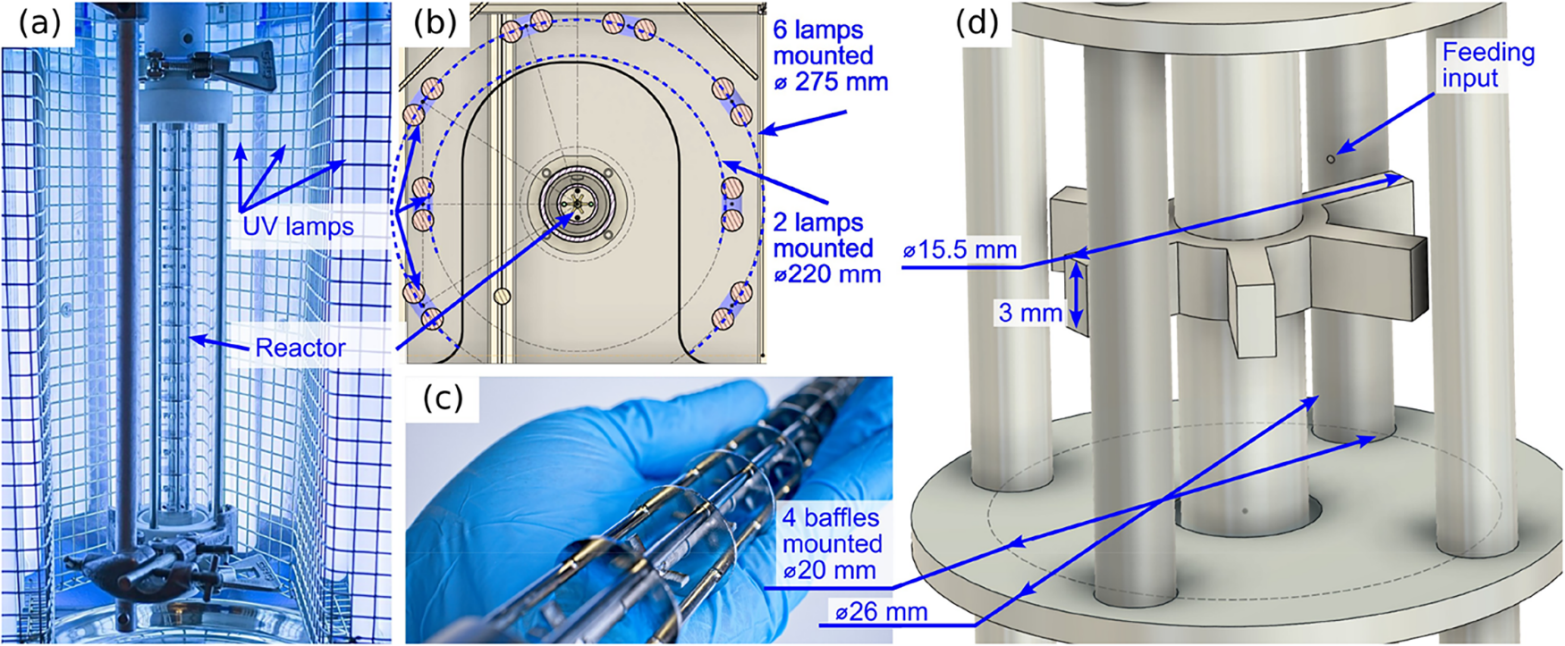
(a) General view of the reactor and lamps, (b) lamp positioning cross section, (c) reactor insert containing 10 stirred tanks, and (d) scheme of the individual stirred tank.

An Ismatec peristaltic pump was used to deliver the liquid feed to the reactor. Compressed air was regulated by using a Brooks Instrument gas mass flow controller. The gas and liquid streams were mixed in a PEEK tee-piece prior to being entered into the reactor. Unless otherwise stated, the agitation speed was maintained at 500 rpm. The feed solution consisted of ethylbenzene (10 g/L) dissolved in a 75% v/v acetonitrile/water mixture, with catalyst loading varied between 5 and 25 mol % SAS across different experimental runs. SAS strongly absorbs UVA light in the 365 nm region emitted by the Osram lamps used in the study. The photocatalyst’s absorption overlaps effectively with the light source, ensuring efficient excitation under the reaction conditions.

Gas chromatography (GC) analysis was carried out using an Agilent 7830A system equipped with an Agilent HP-5 column (30 m length, 0.32 mm internal diameter, and 0.25 μm film thickness). Gas chromatography–mass spectrometry (GC-MS) was performed on an Agilent 7890 system with a 5975C MS detector. The column was Agilent DB-5 (30 m length, 0.25 mm diameter, 1 μm film).

The experimental design and analysis were performed using Design Expert software (ver. 12.0.1.0, Stat-Ease Inc., Minneapolis).

The separation of the SAS catalyst from the corresponding ketone can be easily achieved using standard workup protocols, which require extraction using water-immiscible solvents, e.g., ethyl acetate. In a typical continuous flow photooxidation of ethylbenzene experiment, the resulting reaction mixture was distilled to remove acetonitrile, while the SAS catalyst, along with the unreacted ethylbenzene and reaction products, remained in water. The aqueous phase was extracted with three portions of ethyl acetate, and the extracted organic layers were combined, dried, and subjected to evaporation using a rotavap to obtain crude acetophenone. The aqueous phase after extraction contains the SAS catalyst, which has potential for recycle.

## 3 Results and Discussion

### 3.1 Optimizing the Process Using Design of Experiments (DoE) Approach

The DoE approach has proven to be an essential and effective statistical tool for the development and optimization of experimental techniques, as it minimizes the number of experimental runs required while maximizing information gain. In this study, the RSM was applied to investigate the influence of experimental variables on the photooxidation of ethylbenzene. The experimental runs were designed using a 2^4^ factorial design with four independent variables: catalyst loading (mol %, X_1_), liquid flow rate (mL/min, X_2_), gas flow rate (mL/min, X_3_), and number of lamps (X_4_), each studied at two levels: low (−1) and high (+1), as detailed in Table 1. The total number of experiments was determined using the formula: 2^*k*^ + 2*k* + 6 = 30, where *k* represents the number of independent variables (*k* = 4). The primary response measured was the conversion of ethylbenzene (%).

**Table 1:** Actual Values for the Four-Factor Central Composite Design

factor	name	units	minimum	maximum	coded low	coded high	mean
*A*	catalyst loading	mol %	5.00	25.00	–1 ↔ 10.00	+1 ↔ 20.00	15.00
*B*	liquid flow rate	mL/min	2.00	10.00	–1 ↔ 4.00	+1 ↔ 8.00	6.00
*C*	gas flow rate	mL/min	40.00	860.00	–1 ↔ 260.00	+1 ↔ 750.00	501.33
*D*	no. of lamps	n/a	0.00	8.00	–1 ↔ 2.00	+1 ↔ 6.00	4.00

A total of 30 experiments were conducted in a randomized order to minimize systematic errors and improve statistical reliability. Table 2 presents a comparison between the experimental responses and the predicted values generated by the DoE optimized model. The model’s predictive performance was further validated by plotting the actual versus predicted conversion of ethylbenzene (EB). Additionally, this model was employed to generate response surface plots, which illustrate the individual and interactive effects of the four independent variables on the conversion efficiency.

**Table 2:** Experimental vs Actual Responses

standard	actual EB conversion (%)	predicted EB conversion (%)	residual
1	26.63	24.75	1.88
2	24.25	23.01	1.24
3	34.97	35.96	–0.99
4	40.30	33.86	6.44
5	18.50	25.95	–7.45
6	26.90	24.75	2.15
7	24.80	27.59	–2.79
8	24.82	24.75	–6.75
9	67.20	32.37	1.92
10	64.19	60.99	3.20
11	34.29	58.20	6.76
12	11.69	10.50	1.19
13	22.34	21.12	1.22
14	29.61	34.03	–4.42
15	13.50	16.60	–3.10
16	60.21	60.99	–0.78
17	63.73	64.32	–0.59
18	35.68	33.24	2.44
19	24.47	24.75	–0.28
20	10.09	24.75	1.34
21	1.41	7.10	–5.69
22	38.10	32.72	5.38
23	50.75	56.98	–6.23
24	8.78	18.43	–9.65
25	21.52	25.15	–3.63
26	26.75	24.75	2.01
27	22.95	12.34	10.61
28	19.69	17.22	2.47
29	11.91	7.47	4.44
30	61.09	63.86	–2.36

Coded model equation

2 $$\%\mathrm{EB con}v\mathrm{ersion}=24.75+2.22A-9.59B+0.7596C+14.19D-1.38\mathrm{AB}+0.3081\mathrm{AC}-1.90\mathrm{AD}-0.2181\mathrm{BC}-3.12\mathrm{BD}-1.28\mathrm{CD}+1.21A^{2}+5.10B^{2}-1.35C^{2}+2.68D^{2}$$

Figure 4a presents a diagnostic plot of the experimental versus predicted values in an XY scatter diagram, demonstrating a strong correlation and suggesting good model accuracy. Figure 4b shows the normal probability plot of residuals, which closely follows a straight line with all residuals falling within acceptable limits, further indicating a good model fit. The conversion of ethylbenzene (EB, %) was modeled as a function of catalyst loading (A), liquid flow rate (B), gas flow rate (C), and number of lamps (D) using analysis of variance (ANOVA) for a quadratic model. Among these, the liquid flow rate (B) and number of lamps (D) were identified as the most significant factors, with *p*-values <0.05, as detailed in Table 3.

**Figure 4:**
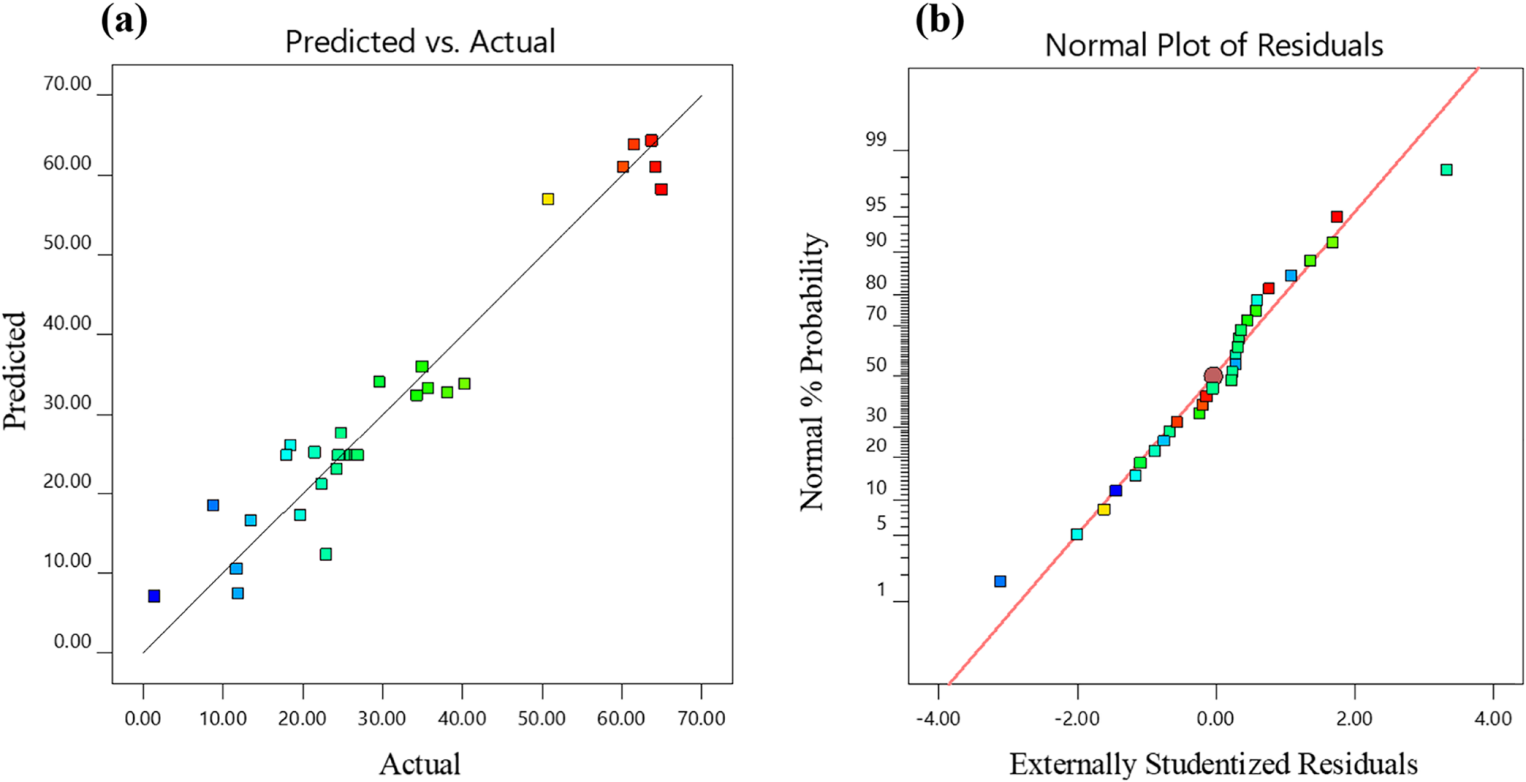
(a) Experimental percentage (%) yield of ethylbenzene against predicted values and (b) normal probability plot of residuals.

**Table 3:** Analysis of Variance (ANOVA) Plot for Response Surface Quadratic (RSM) Model

source	sum of squares	df	mean square	*F*-value	*p*-value
model	8371.34	14	597.95	14.56	<0.0001
*A*-catalyst loading (mol %)	118.33	1	118.33	2.88	0.1102
*B*-liquid flow rate (mL/min)	2208.19	1	2208.19	53.78	<0.0001
*C*-gas flow rate (mL/min)	12.12	1	12.12	0.2953	0.5948
*D*-no. of lamps	4832.26	1	4832.26	117.69	<0.0001
*AB*	30.28	1	30.28	0.7374	0.4040
*AC*	1.52	1	1.52	0.0370	0.8500
*AD*	57.49	1	57.49	1.40	0.2551
*BC*	0.7613	1	0.7613	0.0185	0.8935
*BD*	156.19	1	156.19	3.80	0.0701
*CD*	26.34	1	26.34	0.6416	0.4356
*A* ^2^	40.75	1	40.75	0.9926	0.3349
*B* ^2^	723.27	1	723.27	17.62	0.0008
*C* ^2^	30.89	1	30.89	0.7524	0.3994
*D* ^2^	200.29	1	200.29	4.88	0.0432
residual	615.87	15	41.06		
lack of fit	556.26	10	55.63	4.67	0.0515
pure error	59.61	5	11.92		
cor total	8987.21	29			

A deeper understanding of the interactions between process variables was achieved by graphically representing the regression eq (eq 2) by using 3D response surface plots. These visualizations assist in identifying the optimal values of each factor to maximize ethylbenzene (EB) conversion and overall process efficiency. Figure 5a illustrates the combined effect of catalyst loading (mol %) and liquid flow rate (mL/min) on EB conversion. While increasing both parameters leads to a modest rise in conversion, a plateau is reached at higher values. Notably, the highest conversion is observed at lower liquid flow rates combined with higher catalyst loadings. Figure 5b displays the influence of the number of lamps and catalyst loading. A synergistic effect is evident with EB conversion increasing significantly as both variables are increased. Figure 5c explores the interaction between the gas flow rate and catalyst loading. In this case, increasing either parameter shows only a marginal effect on EB conversion, indicating a weaker interaction. Figure 5d depicts the effect of the liquid flow rate and gas flow rate. Here, a clear trend is observed; EB conversion improves as the liquid flow rate decreases and the air flow rate increases. Figure 5e shows the interaction between the number of lamps and the liquid flow rate. At a lower number of lamps, EB conversion steadily increases with decreasing liquid flow rate. Finally, Figure 5f presents the combined effect of the air flow rate and number of lamps. A consistent increase in EB conversion is observed with simultaneous increases in both parameters, highlighting their positive influence on the reaction outcome.

**Figure 5:**
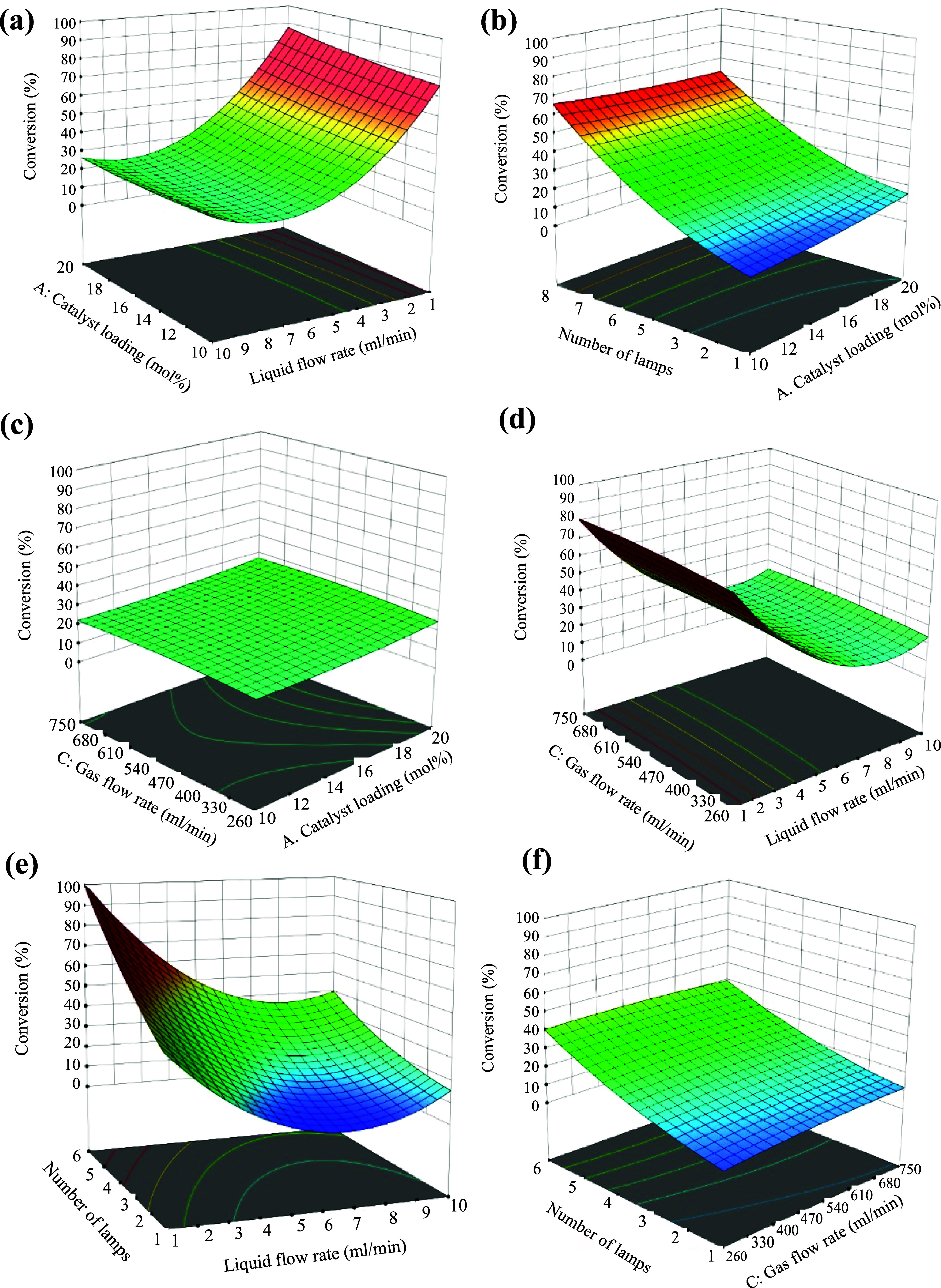
(a) 3D response plot for the effect of liquid flow rate and catalyst loading on EB conversion; (b) 3D response plot for the effect of no. of lamps and catalyst loading; (c) 3D response plot for the effect of gas flow rate and catalyst loading; (d) 3D response plot for the effect of liquid flow rate and gas flow rate; (e) 3D response plot for the effect of liquid flow rate and no. of lamps; and (f) 3D response plot for the effect of gas flow rate and no. of lamps.

### 3.2 Summary of the Optimization Process

The DoE model results (Table 3) identified the liquid flow rate (mL/min) and number of lamps as the most significant factors influencing EB conversion (%). This observation is clearly reflected in the RSM plot shown in Figure 5e, which demonstrates the combined effect of these two parameters on the conversion efficiency. An optimal EB conversion of 63.73% was achieved at a liquid flow rate of 2 mL/min and with 4 lamps, an outcome that would have required extensive trial-and-error experimentation using a traditional one-variable-at-a-time approach.

The response surface plots in Figure 5 collectively reveal a well-defined optimum range for EB conversion, illustrating complex interactions among the variables that could not be captured through sequential experimentation. This systematic approach enabled a more comprehensive understanding of the reaction mechanism and parameter influence.

Overall, the application of DoE and RSM in this study proved to be a powerful tool for both screening and optimization, significantly reducing experimental effort while delivering robust scientific insights into the process behavior and efficiency.

### 3.3 Counter-Current Flow

To explore further enhancement of reactor throughput, the system was configured for counter-current flow of the gas and liquid phases (Figure 6). Counter-current mixing is inherently more efficient than cocurrent mixing due to the sustained concentration gradient between the two phases along the length of the reactor, as illustrated in Figure 7. Operating the reaction under counter-current flow conditions resulted in a notable improvement in ethylbenzene conversion, approximately a 10% increase compared to cocurrent flow, as shown in Table 4. This enhancement directly translates into increased yield, demonstrating the effectiveness of counter-current operation in optimizing reactor performance.

**Figure 6:**
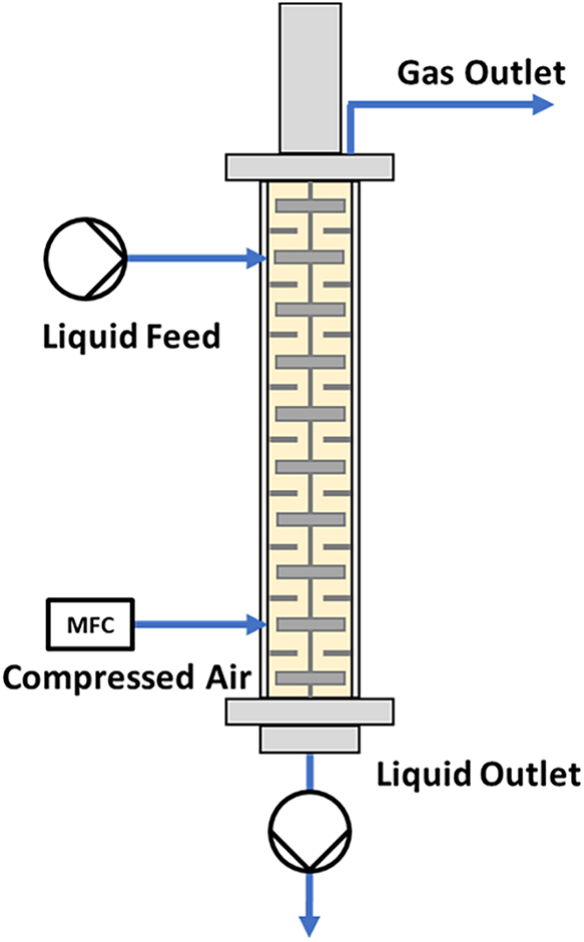
Schematic representation of the counter-current flow setup. Liquid is feed to compartment 9, and gas is feed to compartment 2. The outlet was connected to an additional pump in order to control the flow rate of the liquid leaving the reactor.

**Figure 7:**
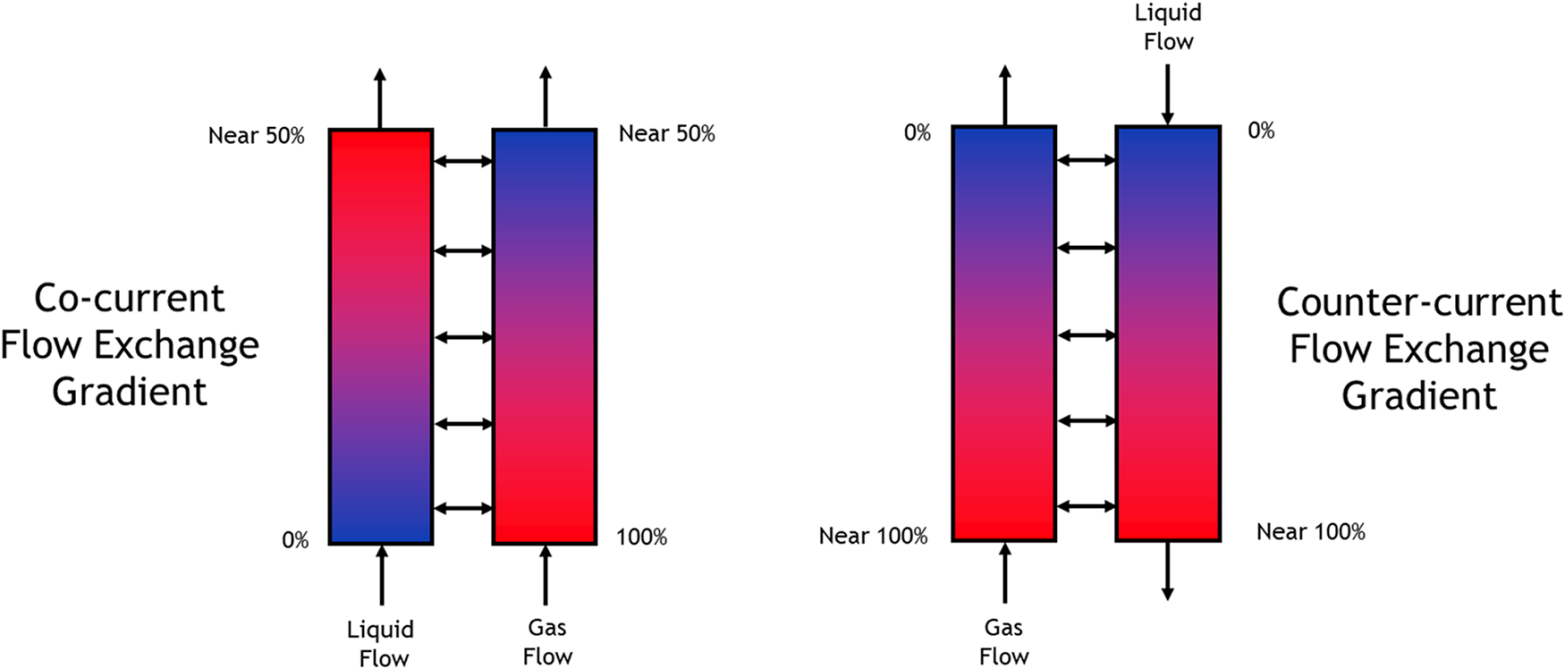
Visual representation of cocurrent flow vs counter-current flow gradient. Moving from a low air concentration (blue) to a high air concentration (red).

**Table 4:** Results Comparing % Conversion, Selectivity, and Yield for Cocurrent and Counter-Current Flow Operation

mode of operation	% conversion	% selectivity	% yield
cocurrent	64.2	81.8	52.5
counter-current	75.5	81.9	61.9

### 3.4 Agitation Speed

The gas dissolution rate is governed by the mass transfer coefficient (*k*_L_ *a*), typically described by eq 4. When all other parameters are held constant, then the mass transfer coefficient (*k*_L_ *a*) and therefore the gas dissolution rate can be enhanced by increasing the total impeller power (*P*_t_).^26^

3 $$\frac{dC}{dt}=k_{L}a\left(C_{sat}-C\right)$$

4 $$k_{L}a=c{\left(\frac{P_{t}}{V_{L}}\right)}^{a}Q_{g}^{b}$$

As shown in Figure 8, increasing the agitation speed from 500 to 750 rpm results in an approximate 6% improvement in both conversion and yield. This enhancement is attributed to improved gas–liquid mixing, which is visually demonstrated in Figure 9.

**Figure 8:**
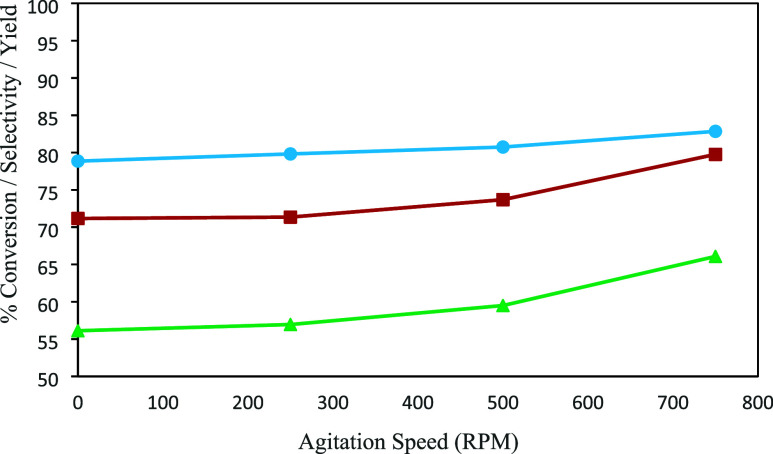
Conversion, selectivity, and yield for varying agitation rates. Red box solid: % conversion; sky blue circle solid: % selectivity; green triangle up solid: % yield.

**Figure 9:**
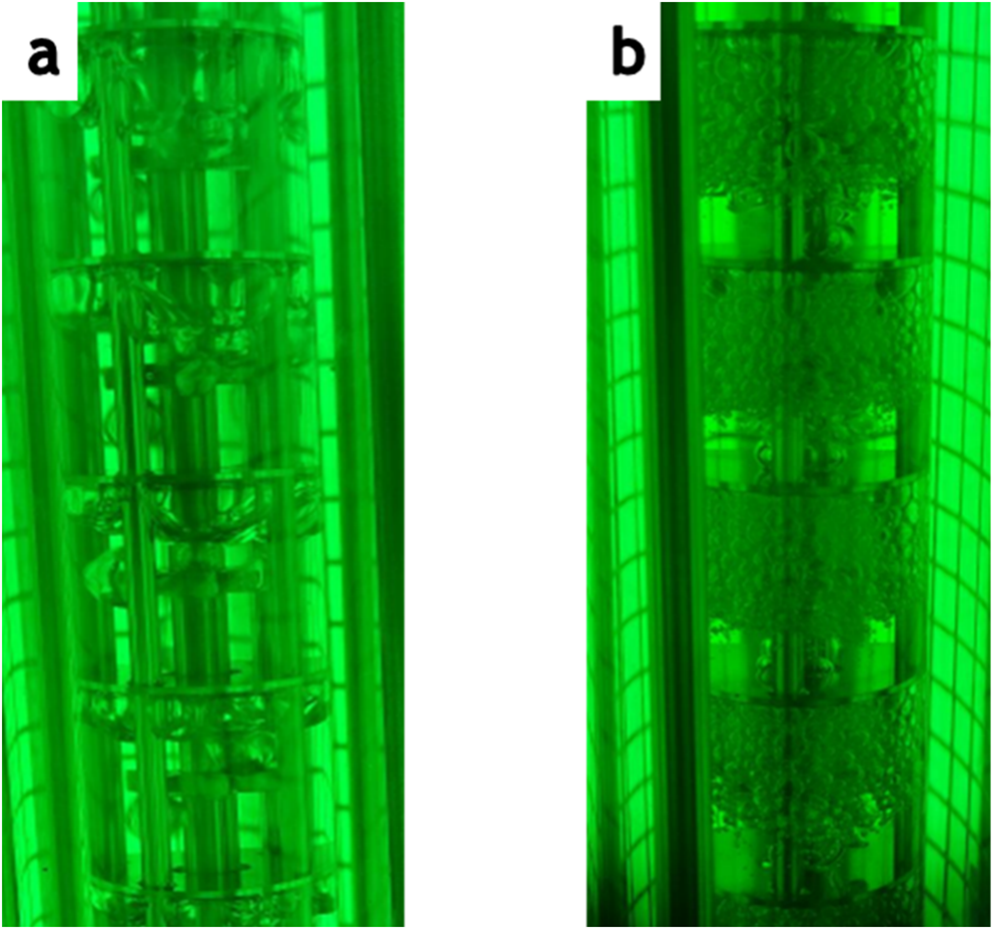
Photographs of gas–liquid mixing at (a) 500 and (b) 750 rpm.

### 3.5 Extended Run

An extended run under counter-current flow conditions was carried out over 8 h to demonstrate process robustness. A 10 g/L solution of ethylbenzene with 20 mol % SAS in a 75% v/v acetonitrile/water mixture was used, processing approximately 1.44 L of solution (equivalent to 14.4 g of ethylbenzene). The liquid flow rate was maintained at 3 mL/min, with a gas flow rate of 260 mL/min and an increased speed of agitation set to 750 rpm. Following the reaction, the mixture was concentrated by using a rotary evaporator to remove excess acetonitrile. The remaining aqueous phase was subjected to three successive extractions with ethyl acetate. The combined organic layers were dried over anhydrous magnesium sulfate and subsequently evaporated to remove ethyl acetate, yielding an orange oil. The process afforded an isolated yield of 87% with a final product purity of ≥98%, as determined by GC-FID analysis.

To compare reactor performance quantitatively, the space–time yield (STY) was calculated using conversion and selectivity data from the feed using the equation,

$$\mathrm{STY}\left(gL^{-1}\;h^{-1}\right)=\frac{\mathrm{XS}C_{A0}M_{W}Q}{V_{R}}$$

where *X* is the conversion of ethylbenzene, *S* is the selectivity to acetophenone, *C*_A0_ is the inlet molar concentration of ethylbenzene (mol L^–1^), *M*_W_ is the molecular weight of acetophenone (g mol^–1^), *Q* is the volumetric flow rate (L h^–1^), and *V*_R_ is the reactor volume (L). Using this relation, the SABRe reactor demonstrated an STY of 14.8 g L^–1^ h^–1^, whereas a conventional 20 m long PEEK tube with 1 mm internal diameter, as employed in our previous study,^13^ achieved an STY of 4.98 g L^–1^ h^–1^. This represents a three fold improvement in STY when using the SABRe reactor, highlighting its markedly enhanced productivity compared to the conventional microchannel configuration.

## 4 Conclusions

A versatile and scalable continuous flow SABRe system was successfully employed for the photooxidation of ethylbenzene. Enhanced mass transfer, achieved through counter-current flow operation and increased agitation speed, led to significantly improved conversion, yield, and overall throughput compared to cocurrent operation. The SABRe reactor achieved a space–time yield (STY) of 14.8 g L^–1^ h^–1^, representing a three fold improvement over the conventional microchannel reactor configuration. Response surface methodology (RSM), implemented via a central composite design, effectively identified and quantified the influence of key process parameters, demonstrating the power of statistical optimization in continuous flow systems. Future scalability of the process could be further enhanced through a numbering-up approach, offering a promising route for industrial implementation.

## List of Symbols

*A*: absorbance
ε: molar absorptivity, M^–1^ cm^–1^
*l*: optical path length, cm
*C*: concentration, M
*t*: time, s
*k*_L_: mass transfer coefficient
*a*: gas–liquid interface area
*C*sat: gas saturation concentration, M
*P*_t_: total impeller power, W
*V*_L_: liquid volume, m^3^
*Q*_g_: superficial gas velocity, m/s
*a*: exponent
*b*: exponent
*c*: exponent
